# Switching to Non-daily Pre-exposure Prophylaxis Among Gay and Bisexual Men in Australia: Implications for Improving Knowledge, Safety, and Uptake

**DOI:** 10.1007/s13178-022-00736-5

**Published:** 2022-06-17

**Authors:** Steven P. Philpot, Dean Murphy, Curtis Chan, Bridget Haire, Doug Fraser, Andrew E. Grulich, Benjamin R. Bavinton

**Affiliations:** The Kirby Institute, UNSW, Sydney, Australia

**Keywords:** Pre-exposure prophylaxis, Qualitative, Gay and bisexual men, Dosing, Event-driven, Switching

## Abstract

**Introduction:**

Preexposure prophylaxis (PrEP) dosing options such as event-driven PrEP hold promise to increase PrEP uptake among gay, bisexual, and queer men (GBQM), but their impacts have not yet been realized and uptake by GBQM suitable for PrEP remains slow in countries where it is only considered an alternative option to daily PrEP.

**Methods:**

We conducted semi-structured interviews between June 2020 and February 2021 with 40 GBQM in Australia to understand PrEP dosing behaviors, knowledge, and preferences.

**Results:**

All participants commenced PrEP daily; 35% had ever switched to non-daily PrEP, mostly taking it event-driven. GBQM who preferred non-daily PrEP had infrequent or predictable sex, were concerned about cost given infrequency of sex, and/or wanted to minimize unnecessary drug exposure. Accurate knowledge of event-driven PrEP was poor. However, reflecting concepts underpinning critical pedagogy, having accurate knowledge was supported by access to consistent messaging across clinical, social, community, and public settings. Several participants who switched to event-driven PrEP had condomless sex events in which they were unable to adhere to pills due to unanticipated sex.

**Conclusions and Policy Implications:**

Implementation of comprehensive and consistent education about correct dosing for event-driven PrEP across multiple settings is needed to ensure increased uptake and safe use. GBQM require messaging about non-condom based HIV prevention strategies when they cannot access daily or event-driven PrEP.

## Introduction

Event-driven oral pre-exposure prophylaxis (PrEP) (also known as the ‘2–1-1’ method) is effective at preventing HIV in cisgender gay, bisexual, and queer men who have sex with men (GBQM) and provides an alternative to daily oral PrEP (Glidden et al., 2016; Hoornenborg et al., 2019; Molina et al., 2015). It involves taking pills for a particular sex event (two pills 2–24 h before sex and two pills for 2 days after sex). Event-driven PrEP is recommended by the World Health Organization (World Health Organization, 2019) and is included in PrEP prescribing guidelines in several countries (Rutstein et al., 2020). These recommendations include guidance on how a person should commence daily PrEP, which involves taking two pills 2–24 h before sex and continuing it daily thereafter (ASHM, 2019; Rutstein et al., 2020).

Globally, most (typically about three-quarters) of those on PrEP take and prefer daily PrEP (Broady et al., 2021; Coyer et al., 2020; Hoornenborg et al., 2019; Jongen et al., 2021; Reyniers et al., 2018; Vuylsteke et al., 2019). However, some PrEP users, when given the choice at study enrolment, chose event-driven PrEP over daily PrEP (Wu et al., 2021; Zhang et al., 2021). Other studies have found that non-users of PrEP would hypothetically prefer event-driven PrEP over daily PrEP (Hall et al., 2016) or that interest in event-driven PrEP was growing (Broady et al., 2021). Studies from Belgium, the Netherlands, and Taiwan, countries in which potential PrEP users are actively provided with a choice of event-driven PrEP by care providers (rather than it just being an alternative), have found that between 21 and 34% of participants have switched to event-driven PrEP, and that uptake of event-driven PrEP is high (Coyer et al., 2020; Jongen et al., 2021; Wu et al., 2021).

In comparison to daily PrEP, event-driven PrEP is attractive to GBQM who engage in infrequent HIV risk, can anticipate when they will engage in risk, or have fewer sexual partners (Carneiro et al., 2021; Chan et al., 2021; Cornelisse et al., 2019; Coyer et al., 2020; MacGibbon et al., 2019; Molina et al., 2017; Reyniers et al., 2018; Vuylsteke et al., 2019; Zimmermann et al., 2019, 2020), experience difficulties adhering to a daily dosing regimen (Cornelisse et al., 2019; Zimmermann et al., 2019), and who have an aversion to medication side-effects, perceived toxicity, or taking more drugs than needed (Carneiro et al., 2021; Cornelisse et al., 2019; MacGibbon et al., 2019; Molina et al., 2017; Zimmermann et al., 2019).

In Australia, rapid dissemination of PrEP in implementation clinical trials began in 2016; the largest of these trials recruited almost 10,000 people in New South Wales and gave free PrEP to all participants (Grulich et al., 2021). On April 2018, PrEP was approved for public subsidy through Australia’s Pharmaceutical Benefits Scheme, providing PrEP for approximately AU $40 per bottle to those with access to Australia’s universal healthcare system, Medicare (Hunt, 2018). Event-driven PrEP and commencing daily PrEP with two pills 2–24 h before sex were endorsed in Australia’s PrEP prescribing guidelines on September 2019, well after daily PrEP was endorsed (ASHM, 2019). Previous PrEP guidelines were cautious and recommended GBQM take PrEP 7 days prior to sex and 28 days after to be protected (Wright et al., 2017). While there has been some promotion of event-driven PrEP from HIV and community health organizations in Australia, this was only recently scaled up in early 2021 (Ending HIV, 2021).

Among GBQM who are eligible for PrEP in Australia, awareness was high at 87.1% in one study, but among those eligible and aware, only 45% used PrEP (Holt et al., 2020). Knowledge of event-driven PrEP appears to be poor — about one-third of PrEP-experienced GBQM have reported prior knowledge of event-driven PrEP (Cornelisse et al., 2019). Critical pedagogy suggests that increased knowledge of an issue does not simply occur through the direct transmission of knowledge from traditional one-way instruction. Rather, increased knowledge occurs through settings that are both vertical (from formal authority sources such as clinicians, formal health promotion on websites, and/or public advertising) and horizontal (through informal social connections and interactions with peers) (Bernstein, 1996; MacGibbon et al., 2021; Southgate & Aggleton, 2017). In some of this paper, we utilize critical pedagogy as a lens for understanding how knowledge of event-driven PrEP can be increased in Australia.

Research into PrEP dosing among GBQM in Australia is limited and focused on quantitatively asking GBQM about their hypothetical interests or preferences (Chan et al. 2021; Cornelisse et al., 2019; MacGibbon et al., 2019). Currently, there is limited research in Australia describing how GBQM tangibly utilize different PrEP dosing strategies over time, including how they value and understand them. Increased understanding of PrEP dosing practices among Australian GBQM will help guide targeted interventions that seek to ensure effective PrEP dosing. Better understanding of GBQM’s PrEP dosing practices also has implications for meeting the PrEP uptake promises not yet realized by event-driven PrEP. In this study, we draw on 40 semi-structured interviews with PrEP-experienced GBQM in Australia to examine PrEP dosing knowledge, preferences, and practices, with a specific focus on non-daily forms of PrEP.

## Methods

Semi-structured interviews were conducted with 40 PrEP-experienced GBQM living in Australia between June 2020 and February 2021. Ethics approval was received by the UNSW Human Research Ethics Committee (HC200377) and ACON (202018).

### Recruitment

To be eligible to participate in the study, men needed to identify as a man (inclusive of trans men), identify as gay or bisexual or have had sex with at least one man (inclusive of trans men) in the previous year, be at least 18 years of age, have a self-reported HIV-negative status, live in Australia, and be able to participate in the interview in English. They additionally needed to have changed their PrEP use in some way since initiating it, including discontinuing for the foreseeable future, discontinuing and recommencing more than once, switched dosing regimens once or more, or a combination of these. Participants needed to have changed their PrEP use because we were interested in recruiting GBQM who had more complex and less streamlined use over time, in an effort to understand and map those complexities.

Participants were recruited in two ways. GBQM from a previous PrEP clinical trial (Grulich et al., 2021) who had given consent to be contacted for research opportunities were emailed and invited to express their interest. Additionally, a community HIV and LGBTIQ health organization sent emails and created Facebook posts to further recruit GBQM. Eligible participants were then contacted by a member of the research team to arrange an interview.

### Data Collection

Semi-structured interviews were conducted with 40 participants. Interviews were conducted virtually via a video platform, lasted between 45 and 100 min, and were audio-recorded and then transcribed verbatim and de-identified. All interviews were conducted by author 1, an experienced qualitative researcher who identifies as a gay man and who has used PrEP. Interviews explored how and why participants initiated, discontinued, and re-commenced PrEP, and if they had switched dosing regimens, how and why they did so, as well has how sexual behavior changed alongside changing PrEP use. They also explored men’s knowledge of how to take PrEP and the different dosing options available. Knowledge of PrEP dosing options was assessed by directly asking what strategies participants knew of and what their understandings of those strategies were. Any strategies not mentioned by participants were raised by the interviewer, who asked what they knew of those strategies. Participants were asked which dosing strategy they preferred, including why they preferred that strategy specifically in comparison to others, and this was the case regardless of whether they had any practical experience of all dosing strategies.

### Analysis

The analytic framework used was a reflexive thematic analysis (Braun & Clarke, 2019). The process began with a close reading of each transcript to ensure familiarity with the data. Short summaries were written for each interview. Transcripts were then imported into NVivo version 12 for coding. As each transcript was re-read, recurring patterns from the data were categorized into a coding framework organized around common themes identified as analysis progressed. For this paper, data relating to participants’ knowledge, practices, and preferences relating to PrEP dosing are utilized. Analyses were conducted by author 1, and analysis interpretations were relayed in regular meetings to the study team, at which point reflections and ideas were shared. Where there were mismatches in interpretations of the data between the study team, author 1, upon receiving advice and opinions, ultimately decided which interpretations to implement into analysis.

## Results

### Sample

The demographics of the sample are presented in Table 1. Ages ranged from 23 to 71 with a median of 39 (*IQR* = 23.5). The majority (*n* = 37) identified as gay. Most (*n* = 32) were Anglo-Celtic, two were born in Australia but reported Asian ethnicity, and six were born in Asia. Of those born in Asia, four had access to Medicare and two did not. The majority (*n* = 25) lived in the state of New South Wales (NSW) in a metropolitan area, eight lived in regional NSW, four lived in the Australian Capital Territory, and one each in Victoria, Queensland, and Western Australia. The sample was highly educated, with 24 having university education. Most participants (28) accessed their PrEP script from their General Practitioner, nine from a sexual health clinic, and three were still using pills from a clinical trial. The vast majority (34) accessed pills from a pharmacy, three imported PrEP online, and three were using pills from the clinical trial.

**Table 1:** Sample demographics

Demographic	Number (*n* = 40)
Age	
18–25	2
26–30	9
31–35	10
36–45	3
46–55	7
56–65	8
> 66	1
Ethnicity	
Anglo-Celtic	32
South Asia	1
Southeast Asia	2
Northeast Asia	5
Sexual identity	
Gay	37
Bisexual	2
Queer	1
Jurisdictional location	
New South Wales city	25
New South Wales regional	8
Australian Capital Territory	4
Victoria	1
Queensland	1
Western Australia	1
Relationship status	
Single	26
Monogamous	4
Non-monogamous	9
Polyamorous	1
Education	
Secondary	10
TAFE/diploma	6
Undergraduate degree	13
Postgraduate degree	11

### Switching to Non-Daily PrEP

All 40 participants initiated PrEP with daily dosing, many as part of a clinical trial of daily PrEP. In total, 14 participants had ever switched to a form of non-daily PrEP throughout their PrEP use, including eight who switched to event-driven PrEP, three who switched to taking pills for between 7 and 10 days in the lead up to sex (only one of whom took pills after sex), and three who switched to taking four pills per week. The latter two strategies are not consistent with PrEP dosing guidelines in Australia (ASHM, 2019). Taking pills for 7–10 days prior to sex would be effective if a person continued to take PrEP for 2 days after the last sex event (which two participants were not doing). Although taking four pills per week has been shown to provide protection from HIV (Anderson et al., 2012), it is not promoted in Australia because it provides no forgiveness for missed doses. It is an informal strategy (PrEP Access Now, 2021).

Those who switched to event-driven PrEP or taking pills for 7–10 days in the lead up to sex did so by discontinuing daily PrEP during a period in which they were not having sex, and then recommencing with their new strategy for their future sexual encounters. Conversely, rather than discontinuing daily PrEP and later recommencing, the three participants who switched to four pills per week simply changed to taking pills on their chosen days rather than daily. These participants described their choices for which days they took PrEP, including Tuesday, Thursday, Saturday, Sunday (sometimes known as “TTSS” or the “Ts and the Ss”), every second day, and around the weekend (such as Thursday to Sunday or Friday to Monday).

Switches from daily PrEP most commonly occurred after 2 or 3 years of being on PrEP, but for a few occurred as recently as within 6 months and as long as 4 years. In addition to participants’ preferences for non-daily PrEP (described below), there were some circumstances that triggered a switch to non-daily use, including the clinical trial ending (and associated costs of publicly subsidized PrEP), moving interstate or returning from overseas travel (and having less sex while settling in), and COVID-19 restrictions (and fewer sexual opportunities). For example, one participant said:

Everyone was in lockdown and things weren’t happening the way they used to. And I figured, “Well, why am I taking this every day? Because it’s highly unlikely that I’ll be needing this today or tomorrow, or the next day.” It just didn’t make any sense continuing it daily (63 years)

### PrEP Dosing Preferences

#### Daily PrEP

Most participants (*n* = 26) used and preferred daily PrEP. Several reasons for daily PrEP preference were provided (Table 2). However, few who preferred daily PrEP had tried event-driven PrEP, so their preferences were not based on experience.

**Table 2:** Reasons for preferring daily PrEP

Reason	Explanation	Example quote
Spontaneity	Participants were unable to plan ahead for sex or valued spontaneity	I never really plan ahead. It’s pretty spontaneous, so if it happens, it happens. So it’s just safer and easier for me to do it on a daily basis (28 years)
Frequency of sex	Participants had sex frequently	At that time I was having a lot of sex so I may as well take it daily (34 years)
Ease/simplicity	Participants conceived of PrEP as more easily taken as part of a daily routine, or that event-driven PrEP required too much consideration	Because I take medications every morning it was like, why calculate [event-driven dosing] when I can just chuck one in every morning and not have to do the calculations? (25 years)
Certainty/confidence	Participants had built confidence in daily PrEp and conversely did not understand or felt unguided about event-driven PrEP	They’d [health practitioners] overwhelm me with all this information [about event-driven PrEP] that was never formal advice. To date, daily use is the only advice I’ve had that I have full confidence in. I never had a doctor give me formal advice around event-based dosing or other advancements that are on the horizon. So, the reason I’m taking it daily is because I have confidence in the advice that I initially got (32 years)
Symbolic value	Taking PrEP daily serving as a reminder of sexual liberation	It’s actually a ritual and it’s kind of a reminder that I’m sexually active, which is a great kind of positive for me having not been sexually active for a lot of my life (60 years)

#### Non-daily PrEP

The 14 participants who had ever used and preferred non-daily PrEP provided several reasons for their preference (Table 3). All of those who preferred non-daily PrEP had used daily PrEP.

**Table 3:** Reasons for preferring non-daily PrEP

Reason	Explanation	Example quote
Infrequency of sex	Sex was too infrequent to warrant daily PrEP	I thought I don’t want to be taking a medication every day and it seems a little pointless if you’re not being sexually active. So if I don’t need to take it, then I don’t want to take it (49 years)
Planning/anticipation	Participants’ circumstances meant they planned sex in advanced	I have an overseas master who I have to seek permission from before I have sex. And, because he’s overseas in a different time zone, everything is a delay. So I always have a few days warning before I’m able to have sex (59 years)
Cost	Due to having infrequent sex, the cost of taking PrEP daily was not warranted	I decided the cost wasn’t justified and I was having to shift from non-pay to pay*. So I decided I had no choice, really. And so I decided it should only be taken at the minimum I had to and shifted to my new model (54 years)
Toxicity concerns	Participants were concerned about having unnecessary, potentially harmful in their eyes, drug circulating in their body, particularly so given sex was infrequent	I take a lot of pills in my life even though I’m healthy. If you can avoid taking all those, it’s a good thing. I wanna live in a more natural world. If I knew that I’m going through a period where I don’t need cover, then I would take that opportunity to give my body a break from the toxicity. (54 years)

^*^The trial in NSW gave people free access to PrEP and then once it was publicly subsidized it came with a cost

### PrEP Dosing Knowledge

#### Incorrect/Poor Knowledge of Event-Driven PrEP

In five interviews, event-driven PrEP knowledge was not explored. Among the 35 in which it was explored, only 14 participants had accurate knowledge of event-driven PrEP. Among the 21 who did not have accurate knowledge of event-driven PrEP, 13 knew that event-driven PrEP existed but did not know its correct dosing, five had very vague knowledge that a non-daily strategy existed but did not know the name of the strategy, and three did not know PrEP could be taken non-daily. Of the 13 who were aware of event-driven PrEP but did not know its dosing, some were almost correct in their assumptions, believing, for example, that a person should take pills for 3 days after sex. Others were much less accurate, stating for example that a person should take pills for 7 days or more prior to sex to use PrEP event-driven.

#### Poor Knowledge of Commencing Daily PrEP

Accurate knowledge about how many pills were needed to achieve protection when commencing daily PrEP was low (*n* = 10). Even participants who understood the importance of the loading dose for event- driven PrEP did not extrapolate that knowledge to the context of daily PrEP commencement. Most participants drew on now outdated advice received from when they first took PrEP to explain dosing for starting, which ranged from 7 days to 1 month.

#### Reasons for Poor Knowledge: Limited Promotion and Low Perceived Relevance

The timing of data collection in comparison to when event-driven PrEP and the two-pill loading dose for commencing daily PrEP were endorsed in guidelines is an important reason event-driven knowledge was poor. The majority of participants commenced PrEP daily because they initiated PrEP in the context of a clinical trial of daily PrEP. When most commenced PrEP (between 2016 and early 2018), event-driven PrEP was not yet formalized into PrEP guidelines in Australia (which occurred in September 2019) and clinicians were only prescribing daily use. Commencing daily PrEP with two pills 2–24 h before sex was similarly not yet promoted. Previous PrEP guidelines were cautious and recommended GBQM take PrEP 7 days prior to sex and 28 days after to be protected (Wright et al., 2017). By the time of data collection for this study, both event-driven PrEP for GBQM and loading doses for commencing PrEP had received endorsement in clinical guidance. However, public promotion of event-driven dosing had only recently commenced and was not at a large scale when most participants were interviewed. Moreover, though some participants said their clinicians had begun raising event-driven PrEP, others had received no further updates since first commencing and so continued to believe that PrEP should only be taken daily. These circumstances in part explain why the level of accurate knowledge was low. Also, however, many daily PrEP users had never viewed event-driven PrEP as relevant to them and so never pursued learning about it.

She [clinician] did tell me about that. She said that I could take it leading up to a sexual encounter or a hook-up. But again, ‘cause I haven’t used it like that, I haven’t really stored the information. Like if it’s like a few days before and then a week after maybe? (35 years)

Often, these participants called attention to the reasons they preferred daily PrEP for having not pursued event-driven PrEP. Some participants, upon having the topic raised by the interviewer, said event-driven PrEP was a strategy they may explore more closely in the future.

#### Sources of Information: the Benefit of Widespread Access to Information

For participants who did have some knowledge of event-driven PrEP or the role of a loading dose in the commencement of daily PrEP, a range of sources and pathways to gaining knowledge were mentioned. Often, these were spread across different settings and time periods; it was rare that one source of information produced accurate knowledge. Some participants recalled first hearing about new dosing information “in the community” through social or gay media outlets, community organization websites or emails, friendships with other GBQM, or in public advertisements. Others said they first heard about event-driven PrEP or the loading dose for daily initiation from their clinicians or through participation in research studies. Importantly, what contributed to accurate knowledge was a combination of clinician-led conversations as well *as* community and social exposure, with one form of exposure triggering the pursuance of information elsewhere. Some participants initially heard about new PrEP dosing information through community avenues and then raised them with their clinician. Others had clinicians raise these options with them and then recalled seeing advertisements or promotion. For example, after changing clinics, one participant said:

He [new clinician] actually talked to me about, “Are you gonna take it each day? Are you gonna take it around every couple of days? How are you planning to take it?” And at that point I then also saw an article and some information around how it could be now taken at the time of sex. I feel it might have been him saying it and then I’ve then seen an article not long after it has come into my world (41 years)

As indicated by this quotation, it was the widespread availability of information across settings that provided opportunities to learn about and normalize non-daily forms of PrEP into a participant’s repertoire.

There was one other way of gaining knowledge about event-driven PrEP mentioned by a few participants. These participants had concerns about the perceived unnecessary drug exposure given the infrequency of their sexual encounters, which was sometimes accompanied by concerns about the cost of the drug. They raised these concerns with their clinicians or friends they viewed as knowledgeable about sexual health, who were then able to offer event-driven PrEP as an option. For example, one participant said:

I don’t know dates exactly but after having that conversation about my lack of sex with [clinician] and then reading a bit more about it, this person said some studies showed it was the case [that event-driven PrEP worked]. And that’s when I thought, “Okay, well, I’m gonna curtail my PrEP consumption for when it needs to be” (49 years)

### Condomless Sex Not Protected by PrEP

Participants who used non-daily PrEP were mostly protected by their own use of PrEP when engaging in condomless sex with casual partners. However, six had engaged in condomless sex with casual partners when not fully protected (according to guidelines) by their own use of non-daily PrEP, usually on several occasions. This included two who were taking pills for 7–10 days in the lead up to sex but none after and four who were event-driven users but had condomless sex without taking their loading doses prior to sex.

The two who took pills for seven or 10 days prior to sex but none after had incorrect assumptions about building up the concentration of PrEP in the body and what to do after sex. These participants based this regimen upon what they recalled hearing when they first started PrEP. This practice partially reflected the early guidelines that encouraged accumulating drug in the body prior to any potential HIV exposure (although such guidance then stipulated ongoing daily dosing) (Wright et al., 2017).

Four participants had sex events not protected by their own use of event-driven PrEP. These participants did not have an opportunity to access their pills when unexpected sex emerged. To counteract not taking their pills according to the event-driven regimen, they took pills after sex, believing this provided at least some protection. Despite these unanticipated events, they believed the sex they had was too infrequent to warrant switching back to daily PrEP. These participants chose not to use condoms for these non-PrEP protected sex events for the following reasons: they disliked condoms; they got caught in the heat of the moment; and they believed the sex they had was likely to be low risk because they assumed their partners were on PrEP or had undetectable viral load.

I would go to the sauna anyway and have risk exposure and take the pills afterwards. So it went from what I considered to be a water-tight system to a system which was better than no system. This way of using the drug and having sex was fine for me because I’m middle class, I go to the sauna during the day. Most of the guys who go there, we’re in this pool of people who are probably on PrEP. So I think I was probably covered but not from a textbook point of view… So I would go home and take two after which I knew wasn’t really the thing but I thought, “Oh, it’s probably better than nothing”. And then I’d stop again and tell myself I had to be better next time (54 years gay)

This quotation reveals that in addition to drawing on assumptions about partners, a few participants consumed PrEP in incomplete and improvized ways after sex, believing that taking PrEP pills at any point provided some protection.

## Discussion

This study provides unique insight into how Australian GBQM understand and adopt different PrEP dosing strategies, including switching to non-daily forms of PrEP. Most participants used and preferred daily PrEP, citing an inability to plan for sex, finding daily PrEP easier to remember, and/or unfamiliarity with non-daily PrEP as their main reasons for their choice. However, a substantial minority switched to non-daily PrEP. Participants’ dosing preferences, and the proportions of those who had switched to non-daily PrEP must be understood in the context of Australia’s PrEP timeline. Daily PrEP was endorsed and promoted well before event-driven PrEP and participants commenced PrEP as part of a trial of daily PrEP. Australia’s specific context may in part explain why more participants preferred daily PrEP and why only 14 had switched to non-daily PrEP. However, these findings nonetheless reflect previous research that has also found PrEP users mainly prefer daily PrEP (albeit in contexts where it is considered the gold-standard) (Broady et al., 2021; Coyer et al., 2020; Hoornenborg et al., 2019; Jongen et al., 2021; Reyniers et al., 2018; Vuylsteke et al., 2019).

Participants in our study who preferred non-daily PrEP explained that it circumvented problems associated with daily PrEP, including having sex too infrequently to warrant daily PrEP, being able to plan for sex, not wanting to pay the publicly subsidized cost given infrequency of sex, and/or wanting to minimize exposure to unnecessary drugs. Previous research has found that barriers to PrEP uptake among those who have never used PrEP are similar (Hannaford et al., 2018; Holloway et al., 2017; Holt et al., 2020; Kesler et al., 2016; Peng et al., 2018; Philpot et al., 2020). Given that a high proportion of Australian GBQM at high risk of HIV acquisition are not accessing PrEP (Hammoud et al., 2019; Holt et al., 2020), event-driven PrEP provides an alternative to daily PrEP that may encourage further PrEP uptake. Indeed, in settings in which there has been at least some PrEP uptake already, and in settings where event-driven PrEP is promoted equally to daily PrEP, event-driven PrEP may be preferable over daily PrEP among GBQM who have never commenced it (Hall et al., 2016; Wu et al., 2021; Zhang et al., 2021). Our findings provide further evidence that health promotion directly targeting GBQM who experience the above issues may increase uptake.

Even among PrEP-experienced GBQM in this study and in reflection of previous research (Carneiro et al., 2021; Cornelisse et al., 2019), accurate knowledge of dosing for event-driven PrEP, and the “loading dose” approach to commencement of daily PrEP, was poor. However, this finding needs to be understood in the context of the timeline of endorsement of these strategies in Australia. Some participants drew on recollections from when they first initiated PrEP (between 2016 and 2018, before event-driven PrEP and the “loading dose” approach were formalized into guidelines) to explain their approach, stating that PrEP should be taken for between seven days and one month to reach full protection. Such recollections reflect dosing recommendations at the time of their commencement (Wright et al., 2017). While there has been some recent promotion of PrEP dosing from HIV and gay community organizations in Australia (Ending HIV, 2021), this had not occurred at the time these interviews were conducted, and it is perhaps unsurprising that their knowledge was poor given Australia’s PrEP timeline. Nonetheless, there is a continued need to scale up education of both event-driven PrEP generally and the “loading dose” approach to initiating daily PrEP, including in PrEP-experienced GBQM who may benefit from updated information. Increased knowledge of event-driven PrEP more generally may help GBQM who have never used PrEP to initiate it if it is suitable for them. Additionally, however, health promotion efforts should be made to increase knowledge of event-driven PrEP among PrEP-experienced GBQM too. This may provide these GBQM with a greater sense of options if their circumstances should change. Increased knowledge of the “loading dose” approach to initiating both daily and event-driven PrEP may benefit some GBQM who have an outdated belief that they should take more pills than is actually needed to reach protection. Increasing this knowledge may reduce pill intake, pill-related costs, time in between taking PrEP and then having PrEP-protected sex, and perceived barriers to recommencing PrEP.

Increases in accurate event-driven dosing knowledge are important given that, due to unanticipated sex events, there were several instances whereby participants were unable to correctly adhere to event-driven dosing, leading to potential HIV exposure. These experiences support prior research reporting that even if event-driven users have infrequent sex in comparison to daily users, HIV protection may be more suboptimal due to non-adherence (Vuylsteke et al., 2019; Wu et al., 2021; Zimmermann et al., 2019). For example, in one study, 97% of sex acts were covered in participants using daily PrEP but only 67% of sex acts were covered for event-driven users (Vuylsteke et al., 2019). The data presented here provide new insight into how and why some event-driven users may not be protected by their own PrEP use. That is, when unplanned sex events occurred, condoms were not a preferred choice, and participants instead made assumptions about sexual partners’ PrEP or viral load status. Moreover, after condomless sex some participants took incomplete, improvized doses of PrEP after sex. This provides further evidence that it is important GBQM fully understand that correctly dosing for event-driven PrEP is necessary to provide adequate HIV protection.

Finally, increased knowledge of and trust in event-driven PrEP was associated with exposure to PrEP information spanning across a variety of settings. This finding has grounding in critical pedagogy (Bernstein, 1996; MacGibbon et al., 2021; Southgate & Aggleton, 2017). Critical pedagogy suggests that increased knowledge of an issue does not simply occur through the direct transmission of knowledge from traditional one-way instruction. Rather, increased knowledge occurs through settings that are both vertical and horizontal. In this study, both vertical (from formal authority sources such as clinicians, formal health promotion on websites, and/or public advertising) and horizontal (through informal social connections and interactions with peers) axes of disseminating information reinforced one another. When combined they empowered participants to actively research event-driven PrEP across multiple settings, or if not actively pursued then participants were at least able to recognize event-driven PrEP in other settings. Moreover, the more that information about event-driven PrEP was available and seen, the more acceptable and normative it was perceived to be. As such, messaging about event-driven PrEP across multiple settings not only served to increase individual knowledge, but additionally likely bolstered its legitimacy as an increasingly mainstream possibility. Contrarily, a few participants had chosen not to pursue event-driven PrEP because they were skeptical of the informal advice they had received when event-driven PrEP was not widely incorporated into prescribing guidelines. For increased knowledge of event-driven PrEP and indeed future PrEP modalities such as long-acting injectables (Landovitz et al., 2021) to become more widespread, information needs to be disseminated comprehensively and with consistent messaging across multiple settings. There should be a particular focus in health promotion efforts on ensuring the information is not just available, but that it circulates among GBQM peer networks.

## Limitations

The findings from our study may not generalize to other contexts as there is widespread access to low-cost publicly subsidized PrEP in Australia. This is not the case in many other countries, particularly in low-to-middle income countries with limited resources and countries with significant HIV stigma and homophobia. There are several characteristics of this sample that may not apply to other GBQM. We captured limited experiences of non-university educated, non-Australian-born, and non-gay identifying men who have sex with men. Participants were previous participants of a clinical trial of daily PrEP and were therefore part of the earlier, and arguably more motivated, wave of PrEP early adopters. Our study also only included GBQM who had ever changed their PrEP use, so we cannot provide insight into those who have never changed from daily PrEP or who have commenced with a non-daily strategy. Many of the participants who described their preference for daily PrEP had not had experience with any other dosing strategy, so participants’ daily PrEP preferences should be considered in light of their responses being hypothetical rather than based on practical experience of all dosing strategies. Also, participants’ poor knowledge of event-driven PrEP and the ‘loading dose’ strategy for commencing daily PrEP was affected by the timing of data recruitment, which occurred around the same time those strategies received endorsement in clinical guidelines. To obtain more accurate data about dosing knowledge, future studies should consider collecting data when dosing strategies have had time to circulate in the community after receiving endorsement. Although we recruited 40 participants overall, only 14 had switched to a non-daily strategy since commencing PrEP. Some of our findings, particularly regarding preferences for non-daily PrEP and condomless sex acts not protected by PrEP, relate only to this smaller sub-sample, so data saturation may not have been reached. There is cause for further qualitative researcher with larger samples of non-daily PrEP users to gain further insights into their preferences and behaviors. Although during coding several discussions about interpretations of the data occurred with the study team, analyses of the free-text data were ultimately conducted by one author, which may have impacted the interpretation of the finalized analysis.

## Conclusion

We have contributed new knowledge about how GBQM in Australia use and understand PrEP dosing, particularly in relation to event-driven PrEP. Findings showed that most participants had a preference for daily PrEP and had low knowledge of accurate dosing for commencing daily PrEP and for event-driven PrEP generally. Those who used event-driven PrEP mostly did so safely, but there were several instances of condomless sex with casual partners not protected by one’s own use of PrEP, with some holding a belief that taking pills after condomless sex provided at least some protection. There is a need to increase awareness of event-driven PrEP generally and also in particular to increase knowledge of the need to follow the regimen accurately. While better awareness may have benefits for increasing PrEP uptake, accurate knowledge is necessary to ensure that event-driven PrEP is effective. Such education efforts should be disseminated comprehensively and with consistent messaging across clinical, community, and public settings.
